# Coenzyme Q10 improves the *in vitro* maturation of oocytes exposed to the intrafollicular environment of patients on fertility treatment

**DOI:** 10.5935/1518-0557.20200003

**Published:** 2020

**Authors:** Sergio Romero, Ricardo Pella, Ingrid Zorrilla, Paola Berrío, Francisco Escudero, Ygor Pérez, Mario García, Carla Gonzalez, Patricia Orihuela

**Affiliations:** 1 Centro de Fertilidad y Reproducción Asistida CEFRA, Lima, Peru; 2 Laboratorio de Biología Reproductiva y Preservación de la Fertilidad, Universidad Peruana Cayetano Heredia, Lima, Peru; 3 Laboratorio de Endocrinología y Reproducción, Universidad Peruana Cayetano Heredia, Lima, Peru

**Keywords:** IVM, oxidative stress, oocyte, chromosome alignment

## Abstract

**Objective:**

To evaluate the impact of patient follicular environment with oxidative stress on oocyte quality.

**Methods:**

Patients on fertility treatment with either advanced maternal age or endometriosis were asked to donate follicular fluid collected during ovum pick-up. Follicular fluid (FF) was added (20%, 10% and 5%; %V/V) to *in vitro* maturation (IVM) medium with mouse oocytes. Following maturation culture, the oocytes were assessed for meiosis reinitiation. In a second setup, coenzyme Q10 was added to culture medium with FF. In addition to assessing meiotic maturation, a subset of oocytes was assessed for spindle structure and chromosome alignment.

**Results:**

Supplementation of IVM medium with FF of patients of advanced maternal age (with or without antioxidants) did not have an effect on the maturation capacity of mouse oocytes. However, the addition of FF of individuals with endometriosis (without antioxidants) in the two highest concentrations affected oocyte maturation (61.5% & 57.0% maturation) compared with the lowest concentration (89.2% maturation) (*p*<0.05). Supplementation of medium with coenzyme Q10 did not improve the maturation rate of oocytes exposed to the FF of individuals with endometriosis (28.5±13.7%) (*p*<0.05). Nevertheless, preliminary analysis of spindle abnormality and chromosome alignment revealed that oocytes resuming meiosis in the presence of FF of patients with endometriosis displayed aberrant spindle morphology and chromosomal misalignment.

**Conclusion:**

The follicular environment of patients with endometriosis severely affected oocyte (nuclear) maturation. *In vitro* maturation in the presence of coenzyme Q10 appears to be a tool for rescuing oocytes exposed to such follicular environment.

## INTRODUCTION

*In vitro* maturation (IVM) of oocytes is an assisted reproductive technology long used in the animal field as a tool to produce embryos *in vitro*. It has not been widely used in human ART, since it is still considered to produce suboptimal results (Vuong *et al.*, 2019). In humans, IVM is indicated mainly for patients with polycystic ovaries (PCO) or polycystic ovary syndrome (PCOS) on account of its nature (minimal stimulation and no hCG trigger), and the almost inexistent risk of ovarian hyperstimulation syndrome (OHSS), a potentially life-threatening condition (MacDougall *et al.,* 1993; Brinsden *et al.,* 1995). Recent advances on IVM indicate a bright future for this technique and broader utilization by other individuals seeking fertility treatment (Sánchez *et al.,* 2017).

Different factors affect the outcome of ART procedures, many of which have not been investigated in depth yet. Some studies reported that lower antioxidant capacity might affect the development of gametes and oocytes in particular. Other authors observed that the production of oxidative agents such as nitric oxide was increased in infertile patients with endometriosis unable to become pregnant (Singh *et al.,* 2013; Goud *et al.,* 2014). Age is an important factor linked to decreased antioxidant capacity (Eichenlaub-Ritter *et al.*, 2011; Agarwal *et al.,* 2012), a particularly relevant factor in the quality of developing oocytes and embryos (Tarin, 1996; Tarin *et al.,* 1998; Zhang *et al.,* 2006).

Some studies showed that oral administration of antioxidants during fertility treatment protects gametes from oxidative stress damage (Tamura *et al.,* 2008). However, most studies have focused on the systemic effects of antioxidants instead of their effects in the gonads.

In our study, IVM was proposed as a way to alleviate the stressful intrafollicular environment in which the oocytes of a group of patients developed (individuals with advanced maternal age and endometriosis). By isolating the cumulus-oocyte complexes from their natural environment (ovarian follicle), the negative effects of such environment are limited, allowing for a less stressful maturation process in the laboratory. Oxidative stress regulation during oocyte IVM has been associated with improved outcomes (Combelles *et al.,* 2009).

## MATERIAL AND METHODS

### Mouse IVM bioassay

The animals used in this study were housed and bred in accordance with national legislation and with the consent of the Ethics Committee of Universidad Peruana Cayetano Heredia (Project number: 64957).

A standard mouse model was used in IVM. Female F1 C57BL x BALB/c hybrid mice aged 23-25 days were primed with 5mIU/ml PMSG. Oocytes were retrieved in an immature stage (germinal vesicle) from ovarian follicles and collected in Leibovitz’s L-15 medium containing 10% heat-inactivated fetal bovine serum (FBS), 100IU/ml penicillin, 100µg/ml streptomycin (all from Gibco), supplemented with 200µM 3-Isobutyl-1-methylxanthine (IBMX; Sigma) to prevent meiosis reinitiation during handling prior to IVM.

IVM was performed for 18h in medium consisting of α-MEM (Gibco), 3mg/mL bovine serum albumin (BSA), 5ng/mL insulin (both from Sigma), 10ng/mL recombinant epidermal growth factor (r-EGF) (Roche), and recombinant human FSH (Gonal-F^®^, Serono).

### Assessment of meiosis reinitiation

Following culture, the oocytes were mechanically denuded with a mouth-controlled fine bore glass pipette. Meiosis reinitiation was assessed based on the observation of the nuclear maturational stage on a stereomicroscope. Nuclear maturation was scored as GV (Germinal vesicle stage), GVBD (when the GV was not visible), MII (first polar body observed in the perivitelline space), or DEG (when the oocyte was degenerated).

### Collection of follicular fluids

Our study included individuals diagnosed with advanced maternal age or endometriosis seeking fertility treatment. Patients with additional conditions (i.e. hydrosalpinx, non-infectious diseases) or diagnosed with more than one condition were excluded from the study. No exclusion was made on the basis of oral antioxidant administration. Patients gave consent to joining the study. The Ethics Committee at Universidad Peruana Cayetano Heredia approved the study design (Project number: 64957).

Follicular fluid (FF) of the first follicle was collected during ovum pick-up (OPU). The rationale for using FF of the first follicle is that this fluid was less likely to be contaminated by blood cells or flushing medium. Moreover, it is a good representation of the ovarian environment the oocytes are in. Following collection, FF samples were centrifuged at 3000 RPM for 10 minutes. The supernatant was collected and frozen, and heat-inactivated in a water bath at 56ºC for 30min prior to further use.

### IVM in presence of patient’s follicular fluid

Follicular fluid was added to the IVM medium used with mouse oocytes at different concentrations (20%, 10%, and 5%). For purposes of control, cumulus-oocytes complexes were matured under standard IVM conditions (refer to mouse IVM bioassay). Following maturation culture, the oocytes were assessed for meiosis reinitiation.

### Effects of adding antioxidant Coenzyme Q10 to the IVM medium

In an attempt to protect the oocytes from the undesired effects stemmed from exposure to FF, 50µM of coenzyme Q10 was added to the IVM medium of oocytes exposed to follicular fluid in a second setup. Due to patient intake patterns, only FF from patients on oral antioxidant therapy was used in this study. For control purposes, oocytes were also exposed to IVM medium with 20% FF taken from individuals enrolled in our egg donation program.

### Spindle and chromosomal analysis in oocytes

In addition to assessing for meiotic maturation, a subset of oocytes that had reinitiated meiosis (GVBD + MII) underwent preliminary evaluation for spindle structure and chromosome alignment.

The oocytes were fixed and stained as described by Lenie *et al.* (2008). Oocyte spindles were stained by sequentially incubating the oocytes on monoclonal mouse anti-alpha-tubulin (Sigma) and Alexa Fluor 488-polyclonal goat anti-mouse antibody (Molecular Probes). Chromosomes were stained with Nucblue_®_ (Molecular Probes). Antibody dilution and intermediary washing steps were performed with a wash-block solution (PBS containing 0.02% sodium azide, 0.2% milk powder, 2% normal goat serum (NGS), 1% BSA, 0.1 M glycine, and 0.01% Triton X-100). Incubation was carried out at 37ºC. Stained oocytes were viewed on an inverted microscope (Motic) equipped with a fluorescent lamp and appropriate filters; images were recorded with software Motic Images Plus 3.0.

### Statistical analysis

Meiotic maturation in different conditions was assessed with the Kruskal-Wallis test followed by Dunn’s multiple comparison test. Percentage values were arcsine converted before statistical calculation. Comparisons of spindle structure and chromosome alignment were performed with the chi-square square. Statistical analyses were performed using GraphPad Prism version 7. for Windows (GraphPad Software, La Jolla California USA, www.graphpad.com).

## RESULTS

### Effects of FF supplementation on mouse IVM bioassay

The supplementation of IVM medium with follicular fluid of patients with advanced maternal age (on or off oral antioxidant therapy) did not affect the maturation capacity of mouse oocytes.

**Figure 1:**
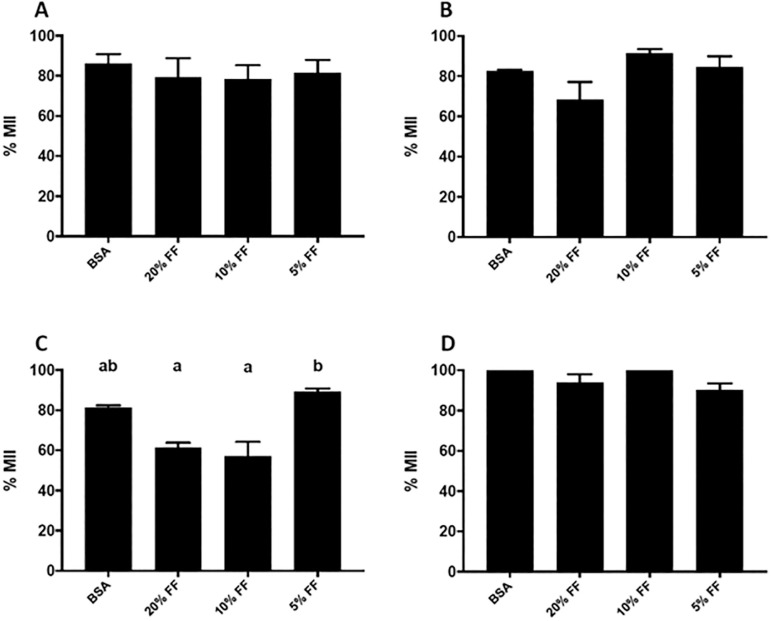
Proportion of maturing oocytes following exposure to patient FF (with and without the addition of antioxidants). A & B, Oocytes exposed to FF of patients of advanced maternal age with and without the addition of antioxidants (A & B, respectively). C & D, Oocytes exposed to FF of individuals with endometriosis, with and without the addition of antioxidants (C & D, respectively). Values are presented as mean±SEM. Different letters denote statistical differences among conditions (*p*<0.005)

Supplementation of IVM medium with follicular fluid of patients with endometriosis in the two higher concentrations significantly affected the maturation of mouse oocytes (61.5% and 57.0% maturation for IVM medium with 20% and 10% FF, respectively) compared with the lowest concentration with 5% FF (89.2% maturation) (*p*<0.05) (Figure 1).

### Effects of adding antioxidant coenzyme Q10 in IVM medium

The addition of antioxidant coenzyme Q10 to IVM medium did not alter the maturation rate (MII) of oocytes exposed to FF of patients of advanced maternal age (AMA).

**Figure 2:**
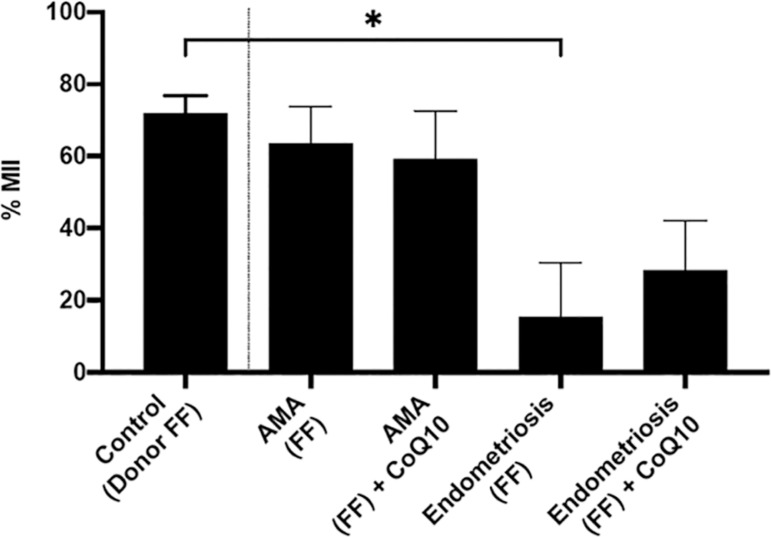
Proportion of maturing oocytes following exposure to FF of patients with or without concomitant incubation with Coenzyme Q10. Values are presented as mean ± SEM. All values were compared to controls (FF of donors from our oocyte donation program). * Denotes statistical difference from control (*p*<0.005)

However, the proportion of MII oocytes was significantly decreased after exposure to FF of individuals with endometriosis (off oral antioxidant therapy) from 71.9±10.1% (Control) to 15.3±30.6% (20%FF). Supplementation with coenzyme Q10 did not significantly improve the maturation rate of oocytes exposed to FF of individuals with endometriosis (28.4±27.4%) (*p*<0.05) (Figure 2).

### Effects on spindle and chromosome alignment

**Figure 3:**
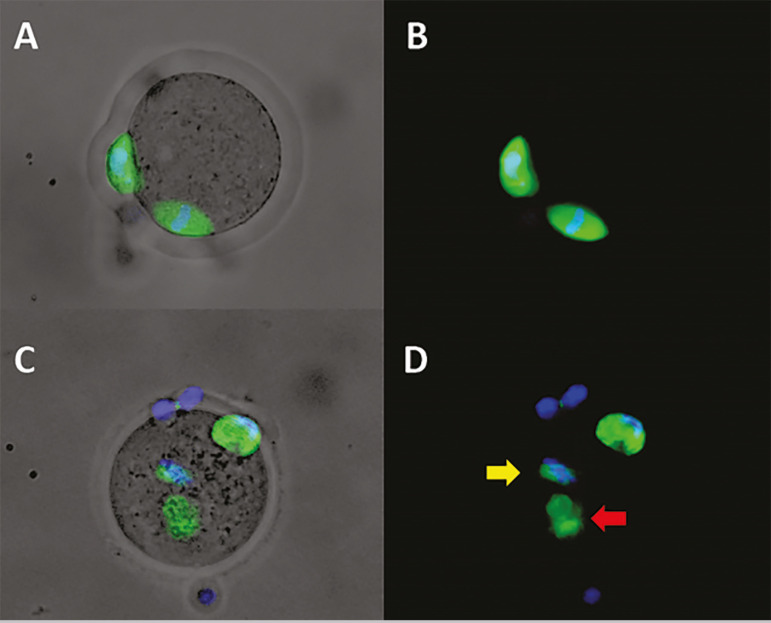
Representative immunofluorescence images of mouse oocytes stained for spindle and chromosomes A & B) MII oocyte, exposed to FF of oocyte donor, with normal spindle morphology and chromosomes correctly positioned in the equator. C & D) MII oocyte, exposed to FF of patient with endometriosis, showing abnormal spindle morphology (red arrow) and chromosomes localized apart from the spindle (yellow arrow). A & C) Composed image of transmitted light + spindle & chromosomal staining. B & D) Composed image of spindle & chromosomal staining.

In order to estimate the impact of stressful culture conditions on chromosome alignment and spindle morphology, chromosomes and spindles were stained for visualization on a fluorescence microscope (Figure 3). The analysis showed that the number of oocytes with aberrant spindles increased after exposure to follicular fluid of patients with endometriosis; however, concomitant exposure to FF of individuals with endometriosis and coenzyme Q10 eliminated such effects (Table 1).

**Table 1:** Spindle morphology and chromosomal alignment of oocytes exposed to patient FF supplemented with Coenzyme Q10

**Condition**	Spindle			Chromosome alignment		
	Normal (n)	Defective (n)	Defective %	Aligned (n)	Misaligned (n)	Misaligned %
Control (Donor FF)	30	1	3.2	27	0	0
Endometriosis FF	6	5	45.5****	6	3	33.3***
Endometriosis FF + Coenzyme Q10	20	0	0	19	0	0
AMA FF	33	1	2.9	34	0	0
AMA FF + Coenzyme Q10	33	1	2.9	32	0	0

FF: Follicular fluid

AMA: Advanced Maternal Age

*** *p*<0.0001

**** *p*=0.0003

No effects were observed on oocytes exposed to FF of patients of advanced maternal age. 

## DISCUSSION

Folliculogenesis is a tightly coordinated process that involves growing oocytes and accompanying granulosa and theca cells. Therefore, changes in follicle and ovary physiology (originated from the likes of disease or hormonal imbalance) may change the behavior of these cells, disturb the follicles, and, more importantly, the oocyte.

Our study looked into the effects arising from two conditions commonly seen in individuals seeking fertility treatment: advanced maternal age and endometriosis. The rationale for focusing on these two groups is that oxidative imbalance has been linked to decreased oocyte quality in patients of advanced maternal age and individuals with endometriosis (Agarwal *et al.,* 2012; Prieto *et al.,* 2012).

Antioxidant capacity decreases with aging (Agarwal *et al.*, 2012). In fact, a review published by Tatone *et al.* (2008) including studies with animal and human models indicated that developing follicles suffer from age-related oxidative stress, most likely due to impairment of antioxidant enzymatic defenses.

Significantly lower MII, cleavage, and pregnancy rates have been reported in individuals with endometriosis (Du *et al.,* 2013), in addition to abnormal oocyte morphology (Borges *et al.,* 2015). However, other authors were unable to find detrimental effects from endometriosis on pregnancy rates (Polat *et al.,* 2014). A meta-analysis reported that individuals with endometriosis had lower fertilization rates and subjects with severe endometriosis (stages III/IV) had poor implantation and clinical pregnancy rates (Harb *et al.,* 2013). Moreover, it has been reported that the follicular fluid of individuals with endometriosis seeking fertility treatment had oxidative imbalance (Prieto *et al.,* 2012; Singh *et al.,* 2013; Goud *et al.,* 2014).

Our results showed that the follicular environment of patients with endometriosis (and not of advanced maternal age) had a detrimental effect on the maturation potential of mouse oocytes. The stressful follicular environment of individuals with endometriosis apparently affects oocyte maturation, as described by other authors (Du *et al.,* 2013).

In regard to spindle morphology and chromosome alignment, our results showed that the follicular environment of patients with endometriosis caused aberrations in oocytes undergoing nuclear maturation. Our preliminary data suggested a potential effect on chromosomal segregation, which might have repercussions on aneuploidy rates of embryos derived from such oocytes, a subject to be addressed in subsequent studies. Furthermore, the addition of an antioxidant in IVM medium seems to protect the oocyte against stressful conditions, suggesting that placing patient oocytes into IVM medium with antioxidants might improve oocyte and embryo quality. This is consistent with the beneficial effects on oocytes of elderly mice of adding antioxidant resveratrol to IVM medium (Liu *et al.,* 2018).

## CONCLUSION

The data presented here supports the idea that the follicular environment of individuals with endometriosis severely affects the maturation capacity of mouse oocytes and indicates that chromosome segregation in maturing oocytes may be impaired. The addition of coenzyme Q10 in IVM medium emerges as a tool to rescue these oocytes.
